# In Situ Preparation of Novel Porous Nanocomposite Hydrogel as Effective Adsorbent for the Removal of Cationic Dyes from Polluted Water

**DOI:** 10.3390/polym12123002

**Published:** 2020-12-16

**Authors:** Badr M. Thamer, Ali Aldalbahi, Meera Moydeen A., Mohamed H. El-Newehy

**Affiliations:** 1 Department of Chemistry, College of Science, King Saud University, Riyadh 11451, Saudi Arabia; aaldalbahi@ksu.edu.sa (A.A.); mabdulhameed@ksu.edu.sa (M.M.A.); 2 Department of Chemistry, Faculty of Science, Tanta University, Tanta 31527, Egypt

**Keywords:** hydrogel, electrospinning, carbon nanofibers, methylene blue, dye removal, adsorption

## Abstract

The use of some hydrogels as adsorbents for pollutants removal from wastewater is limited due to their high swelling properties and the difficulty in recovering them after the adsorption process. To overcome these problems, a new hydrogel nanocomposite based on chitosan-grafted-polyacrylic acid/oxidized electrospun carbon nanofibers (CT-*g*-PAA/O-ECNFs) was prepared by an in situ grafting polymerization process. The prepared hydrogel nanocomposite was used as a novel effective and highly reusable adsorbent for the removal of methylene blue (MB) from polluted water with low cost. The morphology and the structure of CT-*g*-PAA/O-ECNFs were investigated by numerous techniques. The effect of incorporating O-ECNFs on the swelling capability of the prepared hydrogel was explored in distillated water and MB solution at normal pH. The effect of parameters including ratio of O-ECNFs, contact time, pH, initial concentration, and temperature on adsorption process were explored. The adsorption isotherm and kinetic were studied by numerous non-linear models. The obtained results confirmed that the incorporation of O-ECNFs into the hydrogel network decreased the swelling capacity and improved its ability towards MB dye removal. The adsorption process depended on the pH value of the dye solution. Additionally, the adsorption and kinetic results were fitted using the Freundlich isotherm model and pseudo second order model (PSO), respectively. Moreover, the new adsorbents can be recycled for at least five cycles keeping its adsorption capacity and can be easily recovered without loss in its initial weight.

## 1. Introduction

The problem of water pollution is one of the highest challenges facing many countries at the present time, particularly those facing severe shortages in their water resources. The industrial sector engages in water pollution due to the fact that water is used in many industrial processes and they discharge a large amount of it as wastewater [1]. Water pollution can be caused by such main industries such as textile, tanning, and dyeing production. These types of industries depend on water in most of their operations and subsequently release large quantities of water to the environment as wastewater that contains many pollutants [2,3,4]. Since these industries rely mainly on dyes and do not consume them entirely, the wastewater produced from them contains high concentrations of them. Therefore, treatment of wastewater that is produced from these industries before its discharge to the environment is considered an urgent matter to avoid environmental pollution in general and water pollution in particular [5]. Due to the danger of dyes and other pollutants for living organisms, many efforts have been made to find and develop a number of methods for removing and purifying industrial wastewater from various pollutants [6,7,8]. Numerous methods have been used to remove the dyes from polluted water, such as biological methods [9] (e.g., biodegradation and adsorption), chemical methods [10] (e.g., oxidation, photodegradation, and electrochemical destruction), and physical methods [11] (e.g., coagulation/flocculation, filtration, and adsorption). However, the adsorption method is the most common and suitable option for removing many dyes due to its high efficiency, ease of use, and low cost [12,13]. The adsorption method depends mainly on the properties of the adsorbent, which determine the method’s efficiency and the cost. Therefore, the adsorbent should be characterized by good efficiency, abundance, reusability, and low cost. There are several materials that have been used as adsorbents to eliminate several pollutants such as clay [14], metal oxides [15], carbon nanotubes [16], carbon nanofibers [17,18], polymers [19], and metal–organic frameworks (MOFs) [20]. Polymers are considered one of the most important adsorbents due to their abundance and the possibility of chemically modifying them in order to increase their effectiveness and selectivity; as well as the probability of designing them in different forms such as powder, film, membrane, hydrogel, etc. Hydrogels as adsorbents are promising polymeric materials that can be used for dyes removal and other pollutants from wastewater. Hydrogels are characterized by hydrophilicity, their multiple active functional groups, and their three-dimensional porous structure, which contributes to increasing their removal capability [21,22,23]. Although, the hydrogel is characterized by high swelling properties, it has poor mechanical properties after swelling [24]. Their high swelling properties and poor mechanical properties limit their application in treatment of wastewater due to the absorption of large amounts of contaminated water and difficulty of collecting them after the adsorption process [25]. Because of those problems, the probability of using hydrogel as an adsorbent in real wastewater treatment is limited despite its high adsorption capacity. Incorporation of nanofillers containing active functional groups is one of the appropriate ways to reduce the hydrogel absorption of water while maintaining or enhancing its adsorption capacity [26]. There are many materials that have been used as nanofillers for enhancing the properties of hydrogel, such as nano-clay [27], graphene oxide [28], functionalized carbon nanotubes, etc. [29]. For example, Huang et al. incorporated graphene oxide into chitosan-based hydrogel, which resulted in enhancement of its mechanical properties with reducing the amount of absorbed water upon increasing the graphene oxide ratio [30]. Chatterjee et al. incorporated multi-walled carbon nanotubes (MWCNTs) into chitosan hydrogel and used it as adsorbent for Congo red dye removal [29]. They found that the incorporated MWCNTs into hydrogel play an important role in enhancing its porosity and adsorption capacity [29].

Herein, we report the incorporation of oxidized-electrospun carbon nanofibers (O-ECNFs) into chitosan-grafted-polyacrylic acid (CT-*g*-PAA) by in situ polymerization to produce porous hydrogel nanocomposite and their use as effective and highly reusable adsorbent for removal of cationic dyes such as methylene blue (MB). The morphology and physiochemical properties of CT-*g*-PAA/O-ECNFs were investigated by numerous techniques. The effect of O-ECNFs ratio in swelling capacity as well as the adsorption capacity towards the MB dye removal from contaminated water were examined. The adsorption capacity of CT-*g*-PAA/O-ECNFs was evaluated at diverse parameters like pH, temperature, initial concentration, and contact time. In addition, isotherm and kinetic study were analyzed by different non-linear models. Finally, the thermodynamic parameters were calculated, and the recycling of the prepared hydrogel nanocomposite was also studied.

## 2. Materials and Methods

### 2.1. Materials

Chitosan (CT) and acrylic acid (AA) were obtained from Polysciences, Inc., Warrington, PA, USA and QUALIKEMS Fine Chem Pvt. Ltd., Delhi, India, respectively. *N*,*N*’-Methylenebisacrylamide (MBA), ammonium persulfate (APS), and methanol were obtained from Sigma-Aldrich, Darmstadt, Germany. Hydrochloric acid (HCl) and sodium hydroxide (NaOH) were purchased from BDH laboratory supply, UK. Methylene blue (MB) dye was obtained from Loba-Chemie, Mumbai, India.

### 2.2. Preparation of Hydrogel Nanocomposite

**Scheme 1:**
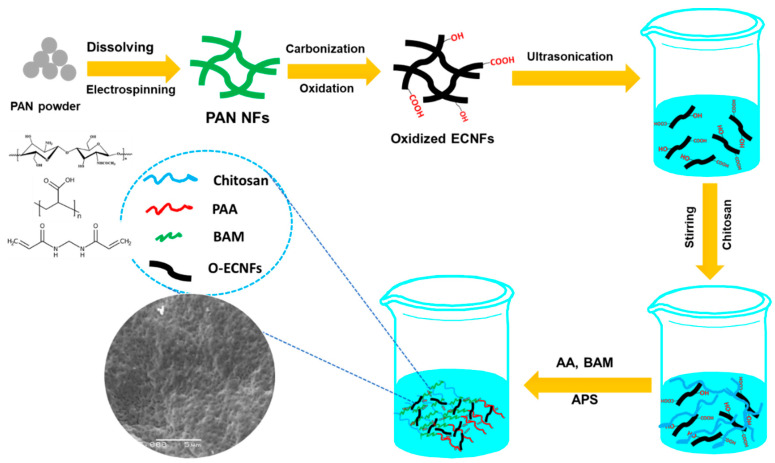
Schematic illustration for the preparation procedure of hydrogel nanocomposite.

O-ECNFs was prepared according to the procedure described by Thamer et al. [31]. A series of CT-*g*-PAA/O-ECNFs nanocomposite hydrogel was prepared by in situ grafting method. In a three-necked conical flask, 0.5 g of chitosan was dissolved in acetic acid (30 mL, 1.0% *w*/*v*) with stirring at 25 °C. After complete dissolution of chitosan, different amounts of O-ECNFs powder (0, 0.5, 1.0, 1.5, and 2.0% *w*/*w* to AA/CT) were dispersed into the chitosan solution by sonication for 15 min and then purged by nitrogen gas for 10 min. Then, the mixture was heated to 60 °C with addition of 0.1 g of APS as initiator under argon atmosphere. After that, AA monomer (4 mL) was added to the mixture at 60 °C with stirring for 15 min, followed by addition of 5 mL of MBA solution (0.097 mol/L) to the reaction mixture with constant stirring (200 rpm) under argon for further 1 h. The reaction product was allowed to cool down to ambient temperature and was neutralized to pH 8 by adding 1 M NaOH solution. To the formed product, methanol (500 mL) was added with stirring. After complete dewatering for 24 h, the hardened superabsorbent nanocomposite particles were filtered, washed with methanol (2 × 50 mL), and vacuum dried at 50 °C. Scheme 1 illustrates the preparation method of O-ECNFs and porous hydrogel nanocomposite The prepared samples were coded as CT-*g*-PAA/O-ECNFs, CT-*g*-PAA/O-ECNFs-0.5, CT-*g*-PAA/O-ECNFs-1.0, CT-*g*-PAA/O-ECNFs-1.5, and CT-*g*-PAA/O-ECNFs-2.0.

### 2.3. Characterization of Hydrogel Nanocomposite

The morphology of hydrogel and hydrogel nanocomposite were investigated by means of scanning electron microscopy (SEM, JEOL2100F, Akishima, Japan). The structure of hydrogel nanocomposite was investigated by Fourier-transform infrared spectroscopy (FTIR, Thermo Fisher Scientific, Waltham, MA, USA) that was conducted from 400 to 4000 cm^−1^ using a KBr pellet. The crystallinity of hydrogel nanocomposite was investigated by X-ray diffraction (XRD, MiniFlex, Akishima, Japan). Thermal stability of hydrogel and its nanocomposite was studied by thermogravimetric analysis and differential scanning calorimetry (TGA and DSC, TA Q500, Pittsburgh, PA, USA) and was carried out under N_2_ atmosphere to study their thermal stability and the effect of the incorporation of O-ECNFs on their thermal behavior. The pore size distribution graphs were obtained using ImageJ software.

### 2.4. Adsorption Study

Initially, the effect of the added amount of O-ENCFs on MB dye adsorption was explored under the following conditions: dosage of adsorbent was 1 g/L, concentration of MB was 1000 mg/L, pH was 7, temperature was 25 °C, and the adsorption time was 24 h. After determining the most appropriate amount of O-ECNFs in the hydrogel, the effect of pH, temperature, initial dye concentration, and contact time on MB dye adsorption process were studied. The pH’s effect was examined at diverse values in the range 3 to 9 and was controlled by a dilute solution of NaOH (0.1 mol/L) and hydrochloric acid (0.1 mol/L). The remaining concentration of the MB dye was determined by UV/Vis spectrophotometer (Lambda 35, Perkin-Elmer, Waltham, MA, USA) at λ_max_ = 664 nm. The effect of initial concentration of the dye in the variety of 500 to 1000 mg/L was studied under these conditions: 1 g/L of CT-*g*-PAA/O-ECNFs-0.5 pH 7, 25 °C, and 24 h. For the effect of time, 0.1 g of CT/PAA/O-ECNFs-0.5 was added to 100 mL of MB solution (1000 mg/L) at pH 7 under agitation at 25 °C. The dye concentration was estimated at different interval times in the variety from 30 to 960 min by taking 20 μL of the dye solution and dilution by distilled water (3 mL), then measuring at λ_max_ = 664 nm. The amount of adsorbed MB dye by the adsorbents at equilibrium (*q_e_*, mg/g) and time *t* (*ԛ_t_*, mg/g), as well as the efficiency of dye removal, were calculated using Equations (1)–(3):

(1) $$q_{t}=\frac{C_{o}-C_{t}}{m}\times V$$

(2) $$q_{e}=\frac{C_{o}-\;C_{e}}{m}\times V$$

(3) $$\%\;Removal\;efficiency=\frac{C_{o}-C_{e}}{Co}\times 100$$

in which, *C_o_* (mg/L) is the initial concentration of the dye solution; C*_t_* (mg/L) is the concentration of the dye solution at time *t*; *C_e_* (mg/L) is the concentration of the dye solution at equilibrium; *V* (L) is the volume of dye solution; and *m* (g) is the adsorbents’ mass.

### 2.5. Reusability of Hydrogel Nanocomposite

To study the regeneration and reusability of CT-*g*-PAA/O-ECNFs-0.5; in 20-mL tube, 10 mg of the adsorbent was added to 10 mL of MB dye (500 mg/L) and was placed on a thermostat shaker water bath for 2 h with a speed of 70 rpm. After adsorption, the hydrogel was removed by tweezer and the residual concentration of MB dye was determined. Desorption process was done by adding HCl (20 mL, 0.1 mol/L) to the adsorbent with shaking for 4 h, followed by washing with distillated water. Finally, the hydrogel was reused after being vacuum dried in oven at 60 °C for 4 h. The adsorption-desorption process was repeated for five cycles.

### 2.6. Swelling Test of Hydrogel Nanocomposite

The swelling behavior of dried pure hydrogel and hydrogel nanocomposite was studied in distillated water and MB dye solutions by adding 15 mg of dried hydrogel into 15 mL of solution and shaking for 4 h at 25 °C. The swelling capacity (g/g) of the hydrogel samples were calculated by Equation (4):

(4) $$Swelling\;capcity=\frac{Wt_{s}-Wt_{d}}{Wt_{d}}$$

where *Wt_s_* and *Wt_d_* are the weight in g of swollen and dried hydrogel, respectively.

## 3. Results and Discussion

### 3.1. Characterization of Hydrogel Nanocomposite

#### 3.1.1. The Morphology of Hydrogel Nanocomposite

**Figure 1:**
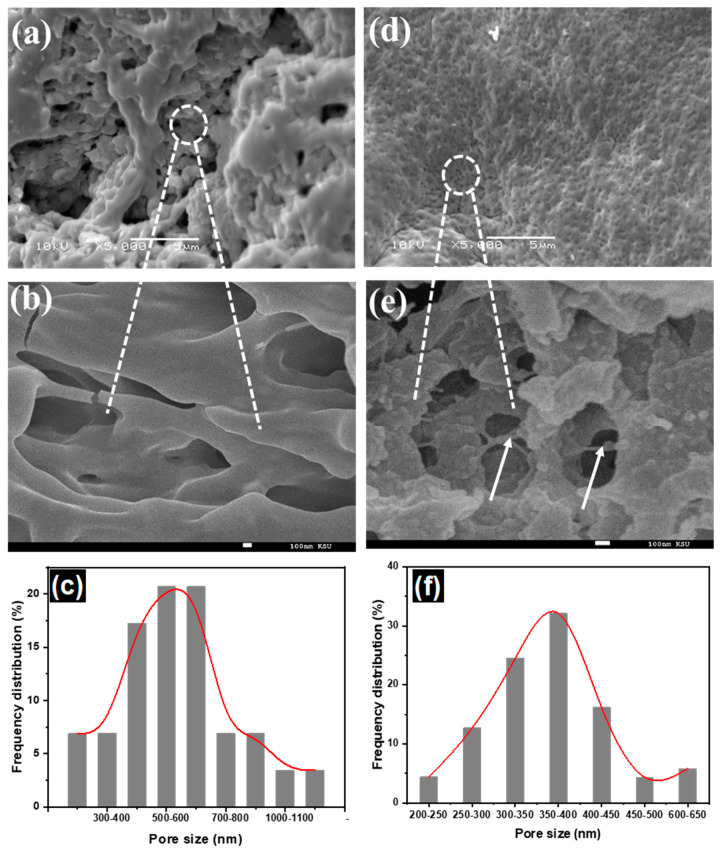
SEM images low and high magnification of (**a**,**b**) chitosan-grafted-polyacrylic (CT-*g*-PAA) hydrogel, (**d**,**e**) chitosan-grafted-polyacrylic acid/oxidized electrospun carbon nanofibers (CT-*g*-PAA/O-ECNFs)-0.5 hydrogel nanocomposite, and pore size distribution of (**c**) CT-*g*-PAA hydrogel and (**f**) CT-*g*-PAA/O-ECNFs-0.5% hydrogel nanocomposite.

The morphology of pure hydrogel and hydrogel nanocomposite was studied by SEM. The surface of dried CT-*g*-PAA hydrogel has three-dimensional (3D) macroporous inner structures with smooth surface (Figure 1a,b), while CT-*g*-PAA/O-ECNFs-0.5 hydrogel nanocomposite has smaller uniform pores with heterogenous surface and more entangled due to the interaction effect of O-ECNFs with hydrogel network (Figure 1d,e). The average of pore size was 712 nm and 353 nm for CT-*g*-PAA and CT-*g*-PAA/O-ECNFs-0.5, respectively (Figure 1c,f). This macroporous structure of hydrogel allows a large amount of solution to pass into the internal structure, leading to high swelling properties. In contrast, the size of the pores of the hydrogel composite was smaller due to the interaction between the O-ECNFs and the hydrogel mesh resulting in reducing the swelling capacity of CT-*g*-PAA/O-ECNFs-0.5. The obtained results are consistent with the study of the swelling capacity of the pure and nanocomposite hydrogels.

#### 3.1.2. FTIR Spectra

**Figure 2:**
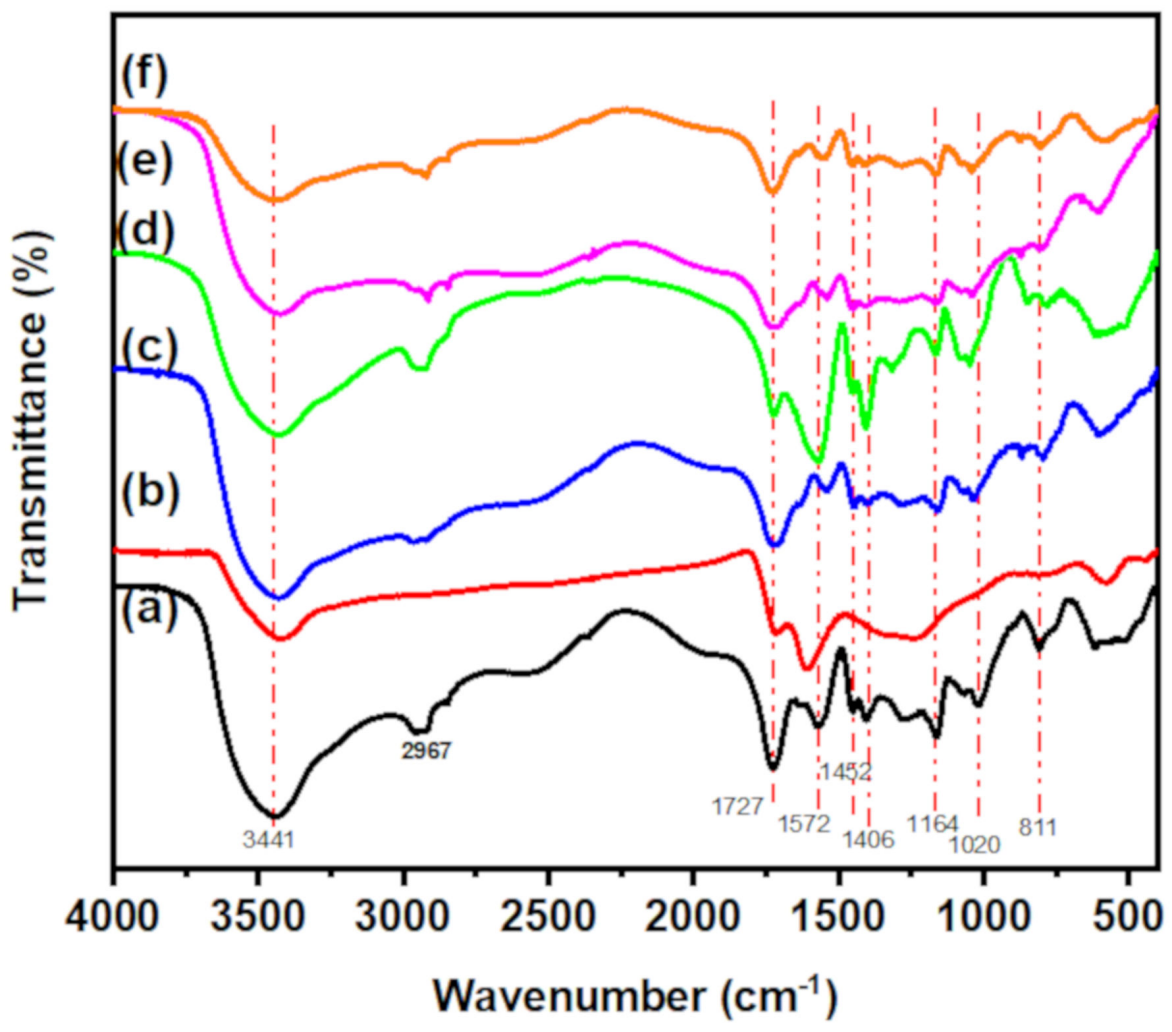
FTIR of (**a**) CT-*g*-PAA, (**b**) O-ECNFs, (**c**) CT-*g*-PAA/O-ECNFs-0.5 (**d**) CT-*g*-PAA/O-ECNFs-1.0 (**e**) CT-*g*-PAA/O-ECNFs-1.5 and (**f**) CT-*g*-PAA/O-ECNFs-2.

FTIR spectra of CT-*g*-PAA hydrogel, O-ECNFs, and CT-*g*-PAA/O-ECNFs are displayed in Figure 2. The CT-*g*-PAA hydrogel exhibits several peaks in the range 600 to 3450 cm^−1^. The characteristic peaks at 3441, 2957, and 2921 cm^−1^ are attributed to –OH stretching and asymmetric/symmetric stretching of -C-H, respectively [32]. Other main peaks at 1727, 1452, and 1406 cm^−1^ are ascribed to -C=O stretching of carboxylic groups and asymmetric/symmetric stretching of –COO^−^, respectively [33]. Peaks at 1164 and 1020 cm^−1^ are assigned to C–O–C asymmetric stretching and C–O asymmetric stretching, respectively [34]. FTIR spectrum of O-ECNFs shows four main distinctive peaks at 3423, 1724, 1615, and 1246 cm^−1^ that are ascribed to -OH stretching, -C=O stretching of carboxylic groups, C=C stretching of graphitic structure, and C-N stretching, respectively [31]. After the incorporation of O-ECNFs into CT-*g*-PAA network, the main characteristic peaks of hydrogel were shifted while increasing their intensity, implying that the functional groups of O-ECNFs interacted with reactive functional groups of the hydrogel via formation of hydrogen bonding [35] and the uniform dispersion of O-ECNFs inside the hydrogel network. The absence of new peaks of CT-*g*-PAA/O-ECNFs is due to the fact that all functional groups of O-ECNFs interacted with the active functional groups of the hydrogel leading to peaks overlap.

#### 3.1.3. XRD Analysis

**Figure 3:**
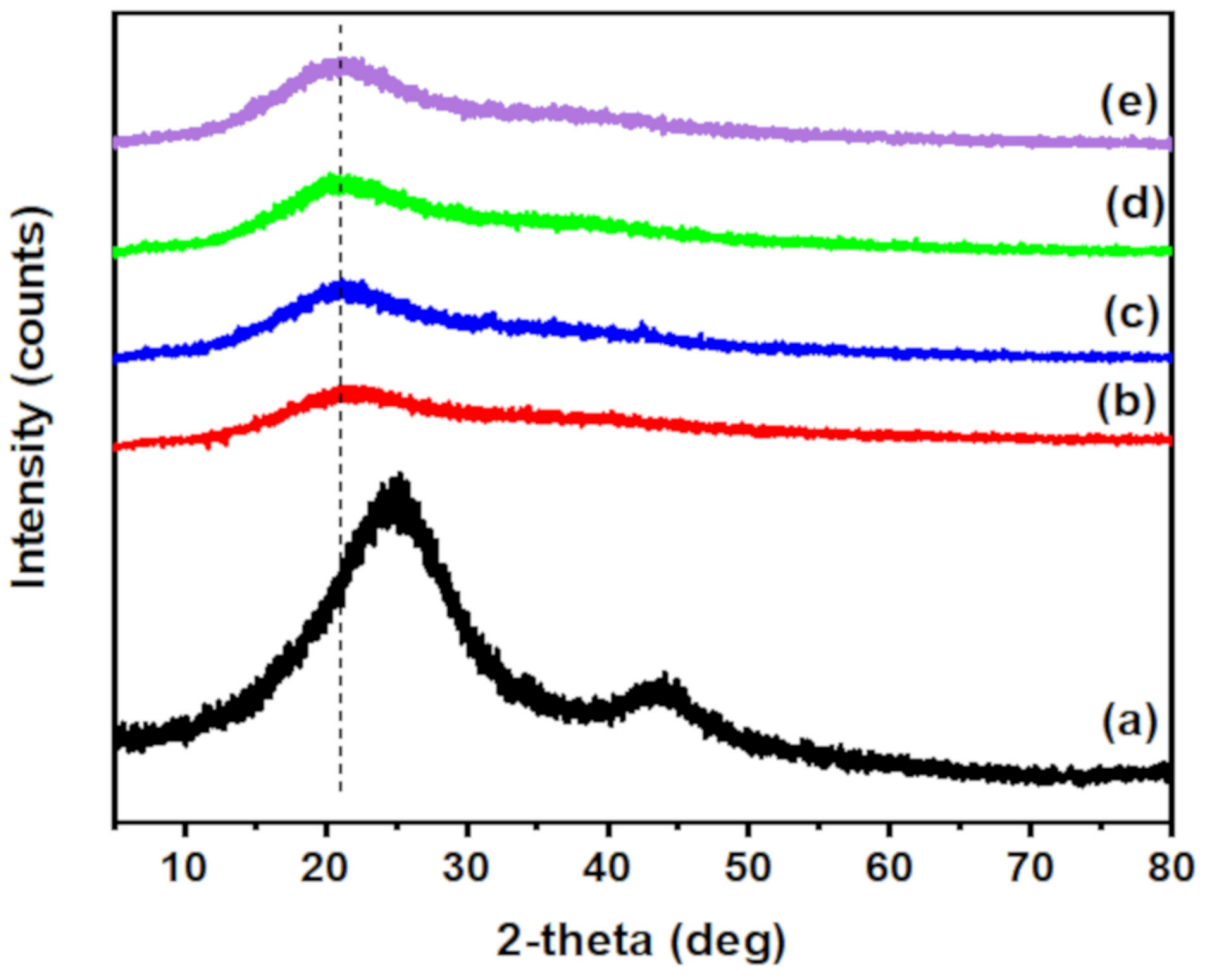
XRD of (**a**) O-ECNFs, (**b**) CT-*g*-PAA, (**c**) CT-*g*-PAA/O-ECNFs-0.5 (**d**) CT-*g*-PAA/O-ECNFs-1.5 (**e**) CT-*g*-PAA/O-ECNFs-2.

XRD is also a valuable method to confirm the incorporation of O-ECNFs and its dispersion in the hydrogel network. The XRD pattern of O-ECNFs displayed two broad peaks at 24.63 and 43.5° that were ascribed to the crystal plane of graphitic layer, and turbostratic carbon plane, respectively (Figure 3a) [36]. The XRD of CT-*g*-PAA hydrogel exhibited only one broad peak at 22.78°, which confirmed its amorphous structure. After incorporating O-ECNFs, the intensity of the main peak increased with slight shift to 21.68° due to the high crystallinity of CT-*g*-PAA/O-ECNFs hydrogel compared to CT-*g*-PAA hydrogel, and the dispersion of O-ECNFs inside the hydrogel matrix was homogenous. Furthermore, the interlayer spacing of hydrogel increased from 0.389 to 0.41 nm after incorporating O-ECNFs, which indicated the intermolecular interaction between the hydrogel and O-ECNFs. These obtained results are compatible with the results of FTIR spectra.

#### 3.1.4. DSC Analysis

**Figure 4:**
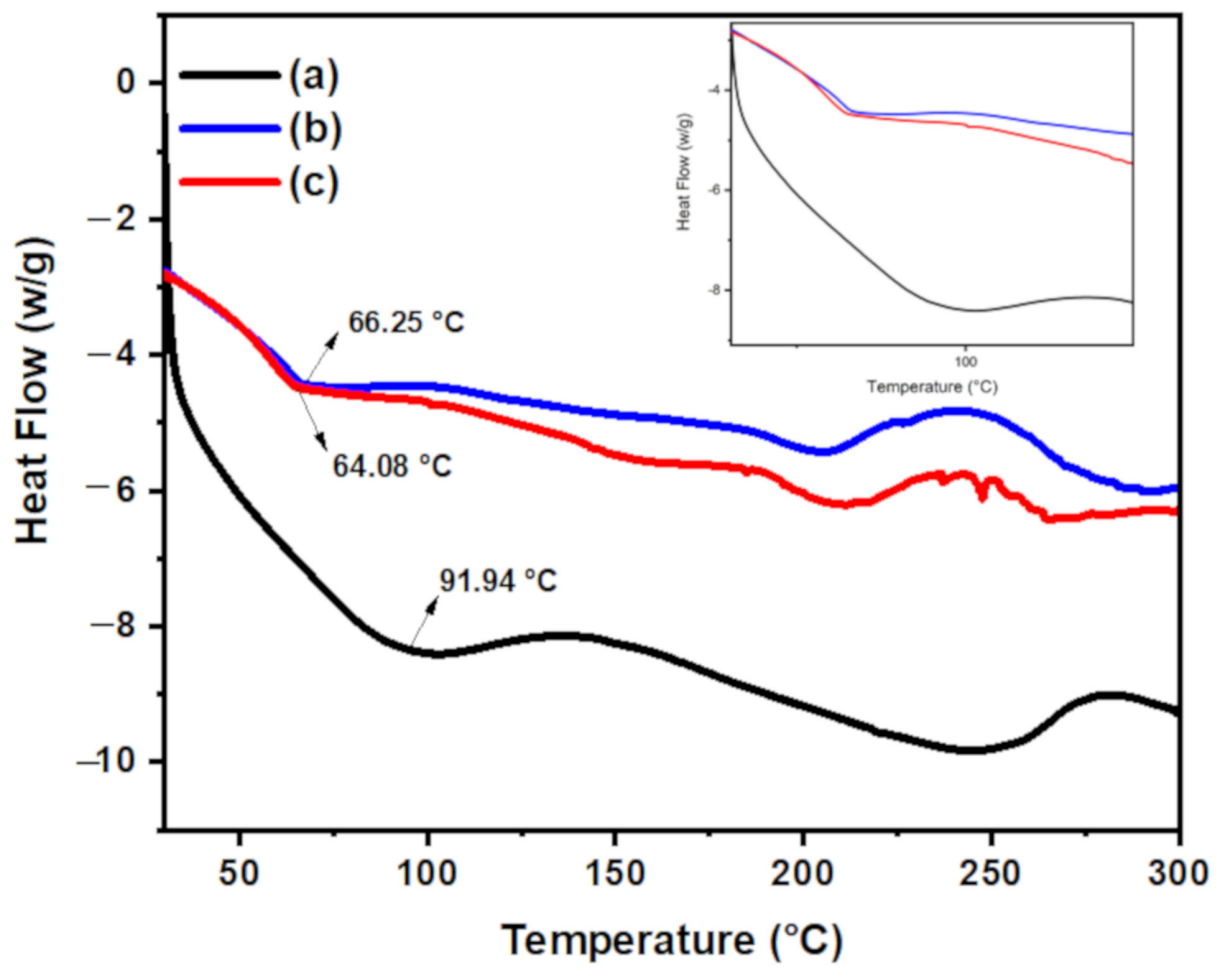
Differential scanning calorimetry (DSC) thermogram for (**a**) chitosan (CT), (**b**) CT-*g*-PAA, and (**c**) CT-*g*-PAA/O-ECNFs-0.5.

The differential scanning calorimetry (DSC) method was used to confirm the interaction between the components of hydrogel nanocomposite. The glass transition temperature (*T*_g_) for chitosan is 91.94 °C and its melting temperature (*T*_m_) is 253.45 °C. Upon grafting of PAA onto chitosan, the *T*_g_ for CT-*g*-PAA shifted to a lower temperature (66.25 °C) and *T*_m_ shifted to 205.77 °C as shown in Figure 4. In addition, upon addition of O-ECNFs to CT-*g*-PAA, the *T*_g_ was further shifted to lower temperature at 64.08 °C and *T*_m_ was observed at 206.56 °C, which may be due to the interaction between free amino functional groups and oxygenated functional groups of CT-*g*-PAA with the oxygenated functional groups of O-ECNFs.

#### 3.1.5. Thermogravimetric Analysis (TGA)

**Figure 5:**
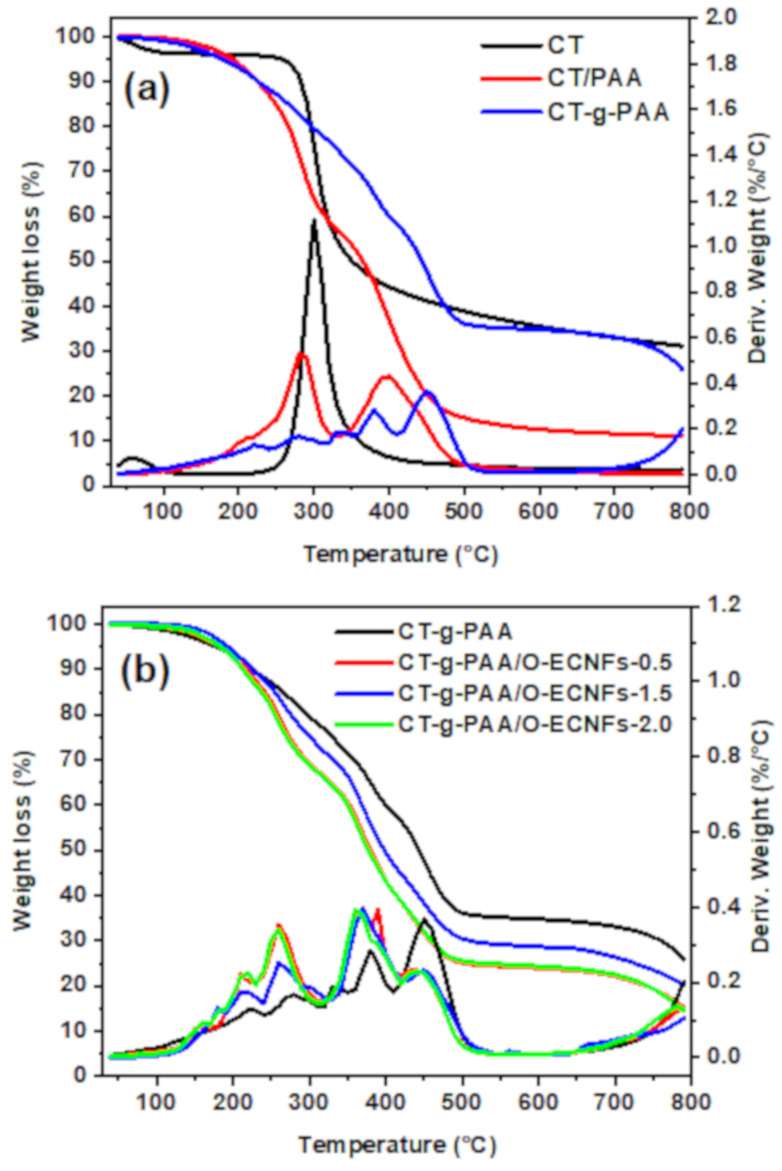
TGA-DTA analysis of (**a**) chitosan (CT), CT/PAA blend, CT-*g*-PAA hydrogel and (**b**) CT-*g*-PAA/O-ECNFs hydrogel nanocomposites.

Thermogravimetric analysis-differential thermal analysis (TGA-DTA) was performed to confirm the grafting and crosslinking process as well as the interaction between O-ECNFs and hydrogel through studying their thermal decomposition. Figure 5a shows TGA-DTA analysis of chitosan (CT), chitosan/PAA without crosslinking (CT/PAA), and chitosan-grafted-polyacrylic acid (CT-*g*-PAA). As shown in Figure 5a, the thermal decomposition of CT proceeds in only one step in the range of 230–443 °C and peaked at 300 °C with residual weight of 31% at 800 °C. In contrast, it was noted that the thermal stability of the CT/PAA was less than CT, and the thermal degradation was done in two main steps at 281 and 395 °C, and the residual at 800 °C was 11.0%. The decrease in the thermal stability of CT/PAA was due to the decarboxylation of the oxygen-rich carboxyl groups. By comparing the thermal stability of CT/PAA and CT-*g*-PAA, the crosslinking improved their thermal stability, and the proportion of the residual weight increased from 11 to 25%. Figure 5b shows TGA-DTA analysis of CT-*g*-PAA hydrogel and CT-*g*-PAA/O-ECNFs hydrogel with diverse ratios of O-ECNFs. The thermal decomposition of CT-*g*-PAA hydrogel takes place in multi-steps, indicating the success of the crosslinking process between the PAA chains and chitosan forming a three-dimensional structure. After incorporating O-ECNFs into hydrogel, the thermal stability decreased due to the deoxygenation of O-ECNFs functional containing groups (-COOH and -OH groups) [31]. These obtained results confirmed the success dispersion of O-ECNFs into the CT-*g*-PAA hydrogel network as well as the physical interaction between their groups via formation of hydrogen bonding.

### 3.2. Swelling Capacity of Hydrogel Nanocomposite

**Figure 6:**
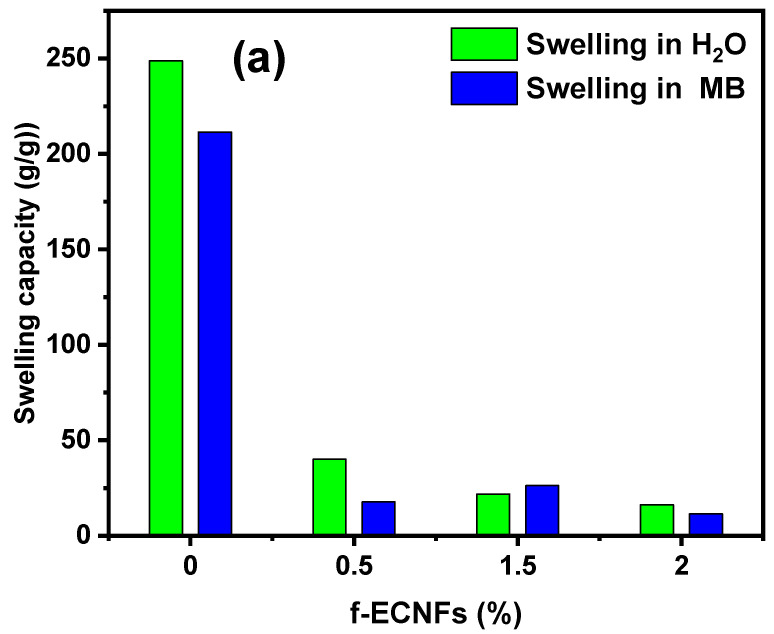

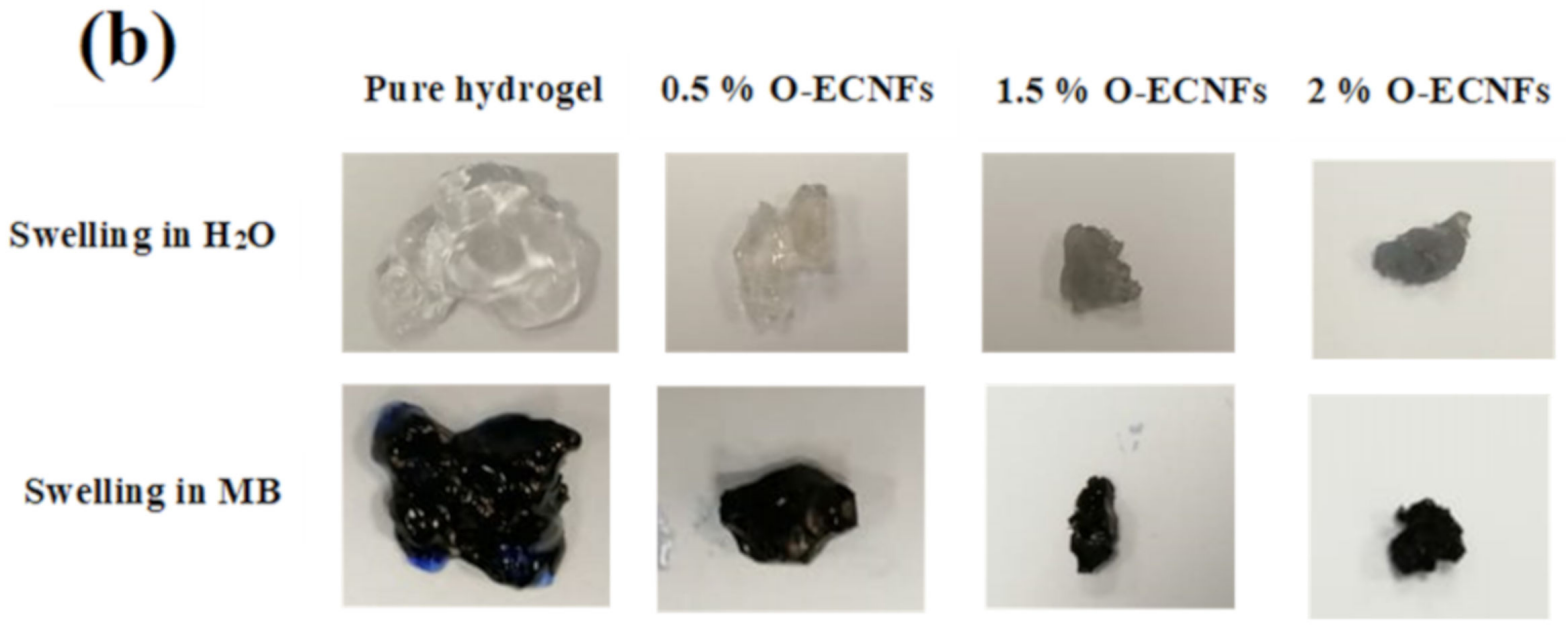
(**a**) Effect of O-ECNFs ratio on the swelling capacity of the prepared hydrogel (**b**) photograph of CT-*g*-PAA hydrogel and CT-*g*-PAA/O-ECNFs hydrogel nanocomposite after swelling in H_2_O and methylene blue (MB).

The hydrogel materials have a three-dimensional structure and also contain polar groups, which enables them to swell and absorb a large amount of polar solvents such as water [37]. The swelling property of the hydrogel with absorption of large amounts of water is of practical benefit in some agricultural applications. However, using the hydrogel to remove pollutants from polluted water is a problem because of its retention of a large amount of water, which reduces the feasibility of reusing the polluted water. Therefore, the control and reduction of hydrogel swelling rate with maintaining its ability to remove pollutants is essential in water treatment. Figure 6a shows the effect of O-ECNFs ratio on the hydrogel swelling capacity in distillated water as well as in MB dye solution. As we can see, the capability of CT-*g*-PAA hydrogel in water and MB dye solution is six and twelve times higher than the hydrogel incorporated with 0.5% of O-ECNFs, respectively. This result clearly indicates the effectiveness of O-ECNFs in reducing the swelling property of the hydrogel through its incorporation into the hydrogel network and the interaction of its carboxylic and hydroxyl groups with the different functional groups of the gel. Interestingly, it was found that the hydrogel swelling capability in the dye solution was less than that of the water solution, which gives the feasibility to reuse the polluted water with dyes. Figure 6b shows a photograph of CT-*g*-PAA hydrogel and CT-*g*-PAA/O-ECNFs hydrogel nanocomposites after swelling in distillated water and MB solution. As we can see, the CT-*g*-PAA hydrogel had a more swollen property than CT-*g*-PAA/O-ECNFs hydrogel nanocomposites and absorbed a large amount of solution. Furthermore, it was noticed that the increase in O-ECNFs ratio from 0.5% to 1.5% led again to slight increase in the swelling capacity in the dye solution, which could be attributed to the increase in hydrophilic groups and the different nature of the dye solution. The increase in the hydrophilic groups led to an increase in the adsorption of the positive dye molecules within the hydrogel network, which led to a slight increase in the swelling capacity due to the electrostatic repulsion between the dye molecules. However, with further increase in the amount of O-ECNFs, the swelling capacity of hydrogel nanocomposite decreased again due to the further increase in the crosslinking density of the hydrogel nanocomposite networks and the aggregations of excessive O-ECNFs in the matrix of hydrogel.

### 3.3. Adsorption Study

#### 3.3.1. Effect of Incorporated Amount of O-ECNFs into Hydrogel

**Figure 7:**
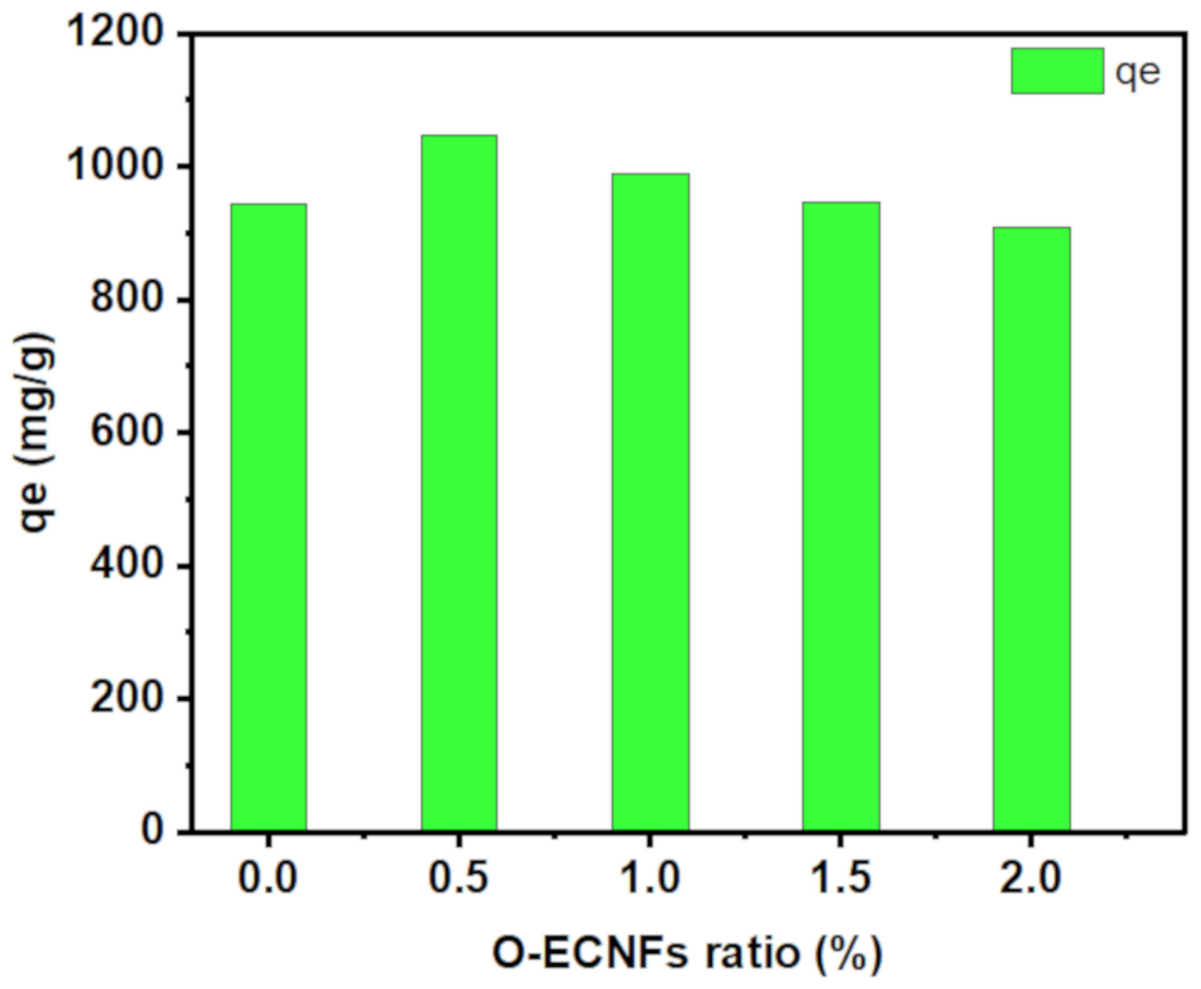
Effect of O-ECNFs amount on the adsorption capability of CT-*g*-PAA/O-ECNFs hydrogel nanocomposites.

The effect of the incorporated O-ENCFs ratio on the adsorption capability of the hydrogel towards MB removal from polluted water was explored. The adsorption capacity of the prepared samples was in the following order: CT-*g*-PAA/O-ECNFs-0.5 (1045.5 mg/g) ˃ CT-*g*-PAA/O-ECNFs-1.0 (989.46 mg/g) ˃ CT-*g*-PAA/O-ECNFs-1.5 (946.19 mg/g) ˃ CT-*g*-PAA/O-ECNFs-0 (943.7 mg/g) ˃ CT-*g*-PAA/O-ECNFs-2.0 (908.34 mg/g) (Figure 7). The great adsorption capability of CT-*g*-PAA hydrogel is due to the existence of the amino and carboxylic groups, which increased their affinity towards cationic pollutants. Moreover, after incorporation of O-ECNFs, the increase in adsorption capability can be ascribed to good dispersion of O-ECNFs in the CT-*g*-PAA network, which provided additional functional groups (-COOH and -OH groups) that enhanced their interaction with cationic MB dye. It is noteworthy that the optimum amount of added O-ECNFs to the CT-*g*-PAA hydrogel was 0.5% (*w*/*w*). Upon incorporation of O-ECNFs into CT-*g*-PAA, the pore size was decreased, resulting in an increase in the adsorption capability of CT-*g*-PAA/O-ECNFs. This result agrees with the result obtained from SEM images. However, with further increase in the amount of O-ECNFs, the adsorption capacity of CT-*g*-PAA/O-ECNFs was decreased again due to the aggregations of excessive O-ECNFs in the matrix of hydrogel [35].

#### 3.3.2. Effect of pH

**Figure 8:**
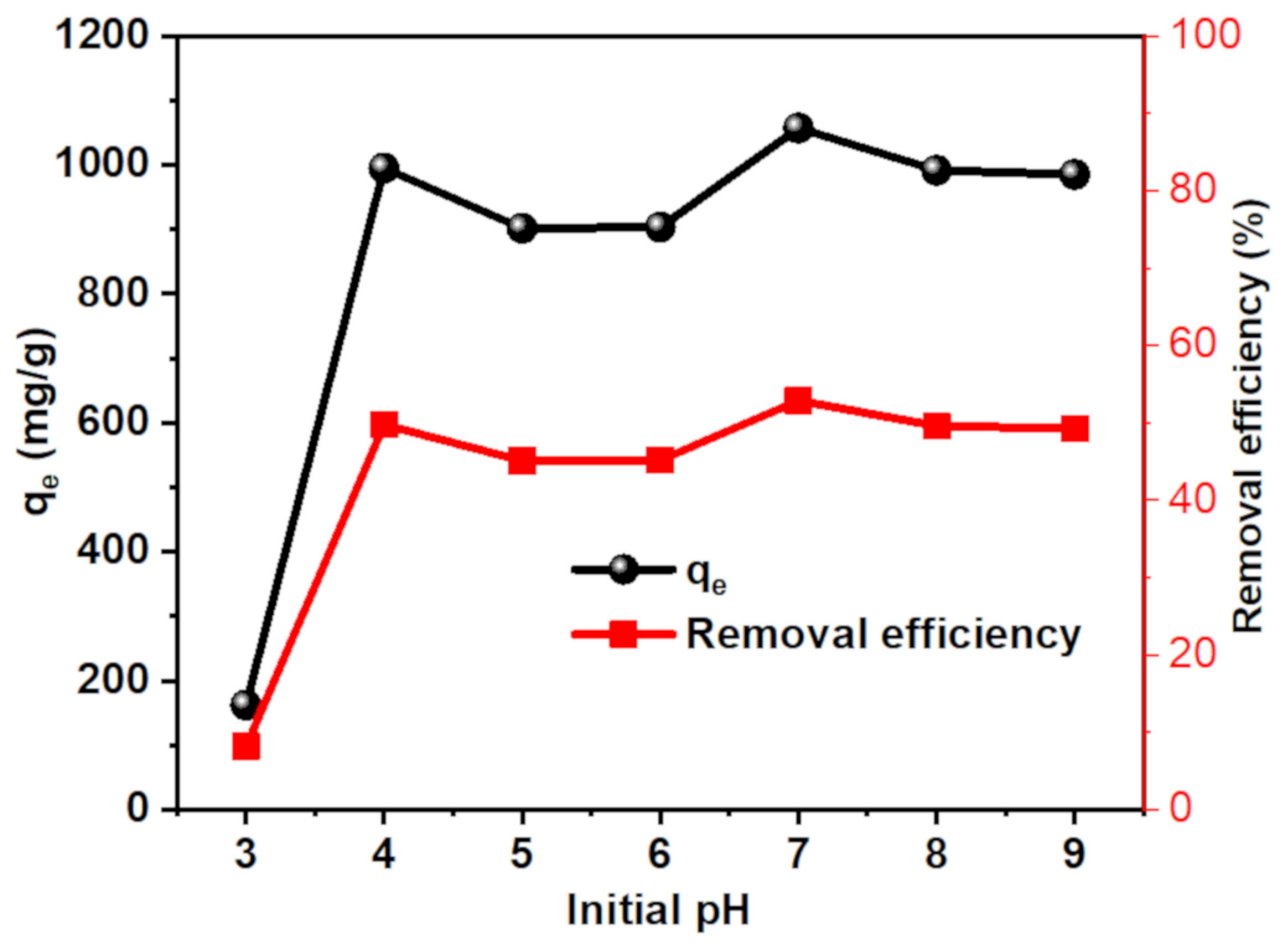
Effect of pH on the adsorption capability of MB by CT-*g*-PAA/O-ECNFs.

The pH plays a significant role in the adsorption process of ionic pollutants on the surface of adsorbents that contain polar functional groups. This effect is ascribed to the ionization of polar groups, whether in the pollutant or the adsorbent. Therefore, the effect of pH on the adsorption capability of MB dye by CT-*g*-PAA/O-ECNFs-0.5 was investigated in pH ranges of 3–9. The adsorption capability increased dramatically with increasing pH value from 3 to 4 and reached the maximum at pH 7 (Figure 8). The sharp decrease in the adsorption capability at pH 3 is due to the protonation of the amine and carboxyl groups, which leads to a repulsion between the positively charged dye molecules with the gel. At pH 4, the carboxylic groups become deprotonated, which is corresponding to its p*K_a_* 4.2, and hence they can interact with MB by electrostatic interaction. Because the pH of the textile wastewater ranges from 5 to 12, the CT-*g*-PAA/O-ECNFs has the ability to remove cationic dyes with high efficiency in a wide pH range [2].

#### 3.3.3. Effect of Initial Concentration and Isotherm Study

**Figure 9:**
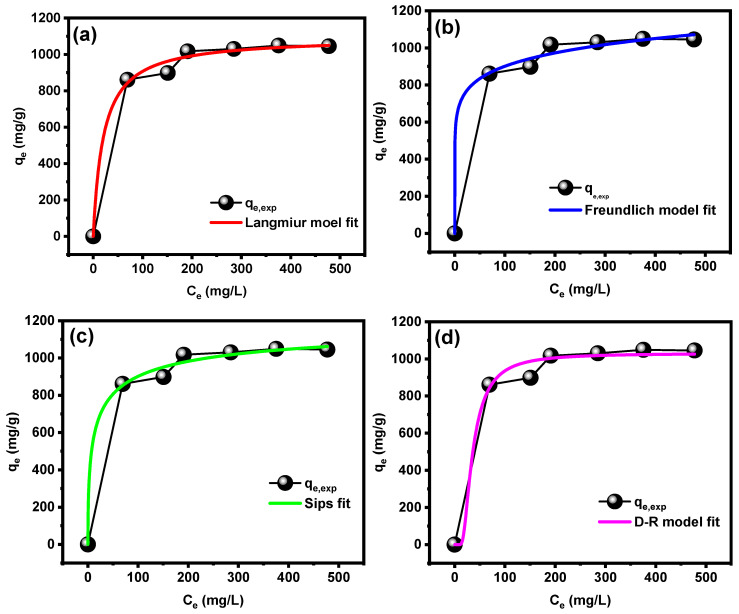
Adsorption isotherms of MB dye onto CT-*g*-PAA/O-ECNFs fitted by (**a**) Langmuir, (**b**) Freundlich, (**c**) Sips, and (**d**) Dubbinin-Radushkevich (D-R) non-linear models.

The isotherm study is an essential study for the exploration of adsorption behavior of the pollutant on the surface of the adsorbent material. Therefore, the adsorption of MB dye was studied by different non-linear models with the purpose of identifying an appropriate model describing the dye adsorption behavior by the CT-*g*-PAA/O-ECNFs. The isotherm models used in this study are Langmuir [38], Freundlich [39], Sips [40], and Dubbinin-Radushkevich [41]. Table 1 summarizes non-linear equations and parameters of all those models. In order to define the model that accurately defines the adsorption behavior of MB dye onto CT-*g*-PAA/O-ECNFs, the relationship between the residual concentration of the dye (*C_e_*, mg/L) versus the adsorption capability (*q_e_*, mg/g) was drawn, and then the fitting was performed by origin pro program as shown in Figure 9. Based on the high value for *R*^2^ and the low value for *χ*^2^, the most suitable model to define the adsorption behavior of MB dye was the Sips model. This obtained result shows that the surface of the hydrogel network is heterogeneous, and the adsorption takes place in multiple layers in reversible process. A value of 1/*n* from the Freundlich model is less than unity and higher than zero, which confirmed that the adsorption of MB dye onto the surface of CT-*g*-PAA/O-ECNFs is favorable. Fitting to D-R model was also fairly good (*R*^2^~0.988 and *q_D-R_* ≈ *q_e_._exp_*) and allowed the calculation of the mean free energy *E* (kJ/mol) of adsorption per molecule of MB. The *E* value is a good indication of whether the adsorption is carried out physically or chemically. Therefore, when the carbon value is lower than 8 kJ/mol, the adsorption is a physical process, and it will be a chemical process when its value is higher than 16 kJ/mol. The value of *E* for adsorption MB onto CT-*g*-PAA/O-ECNFs was 0.056 kJ/mol, which confirmed that the adsorption takes place in a physical process and agrees with the thermodynamic and desorption study. The maximum adsorption capability (*q_o_*, mg/g) was 1030.39 mg/g according to the D-R model.

#### 3.3.4. The Effect of Contact Time and Kinetic Study

**Figure 10:**
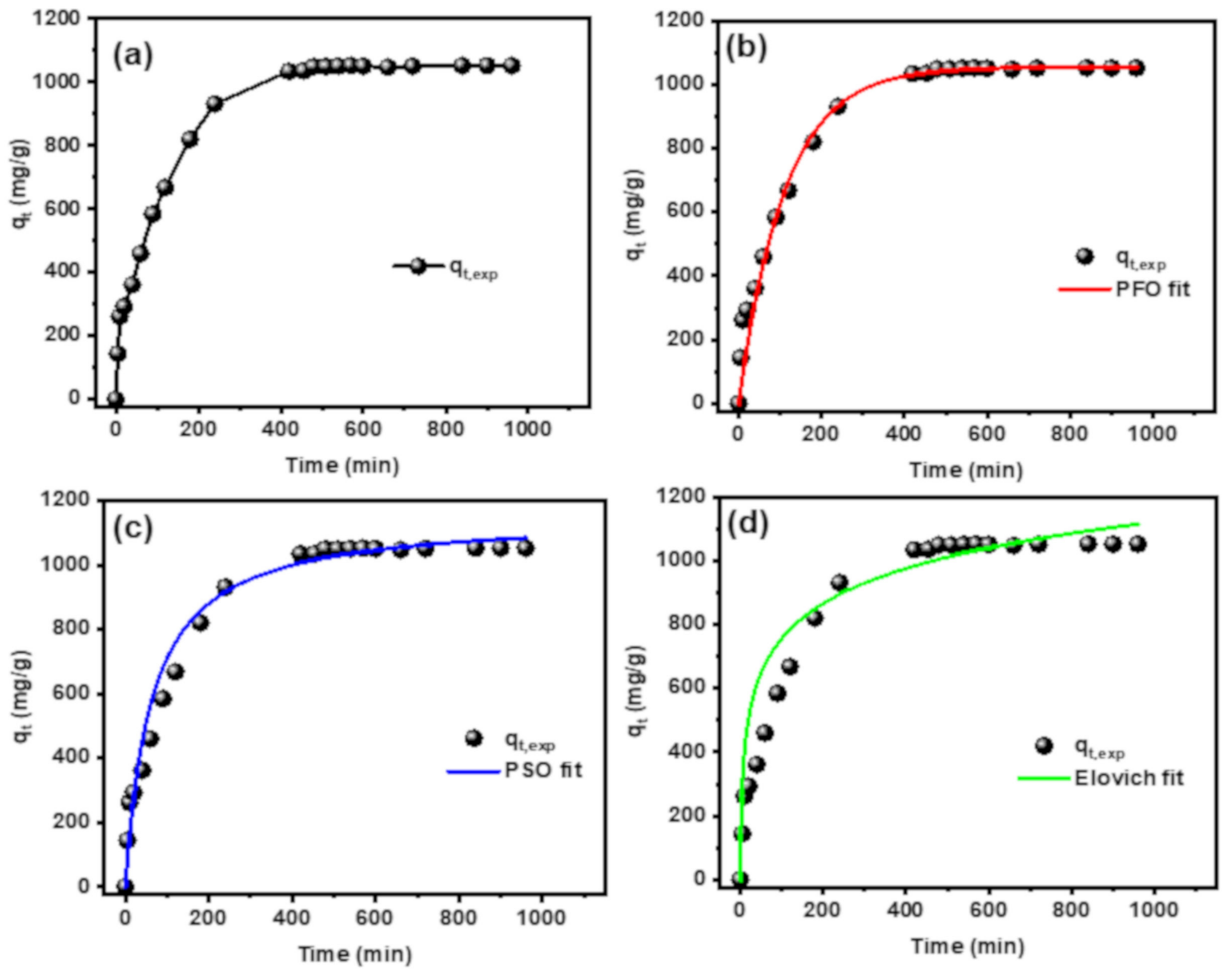
(**a**) Effect of the contact time on MB dye adsorption (**b**) non-linear Langmuir model, (**c**) non-linear Freundlich model, and (**d**) non-linear Elovich model.

The required time for adsorption of MB dye plays an important role in determining the efficiency of the adsorbent with explanation for the adsorption mechanism. The effect of contact time on adsorption of MB dye onto CT-*g*-PAA/O-ECNFs was studied and shown in Figure 10a. The adsorption capacity (*q_t_*) for the removal of MB dye increased sharply within the first four hours and reached to 88.5% of the adsorption capacity at equilibrium state (*q_e_*). With increasing the contact time, the number of MB dye molecules in solution decreased due to their transferring from solution to the hydrogel network until occupying all adsorption sites. The required time to reach equilibrium and saturate the adsorption sites of CT-*g*-PAA/O-ECNFs hydrogel nanocomposite with the dye molecules was seven hours (Figure 10). The adsorption kinetics of MB dye onto CT-*g*-PAA/O-ECNFs was studied by different non-linear models containing pseudo-first-order (PFO) [42], pseudo-second-order (PSO) [43], and Elovich [44] models, and the kinetic plots are displayed in Figure 10b,c. Table 2 summarizes nonlinear equations and parameters of applied kinetic models. The correlation coefficients (*R*^2^) and chi-squared (*χ*^2^) values of the PSO model were higher than those of the PFO model and lower than those of the Elovich model. Therefore, the PSO model provided a good description of the behavior of the MB dye adsorption onto CT-*g*-PAA/O-ECNFs.

**Figure 11:**
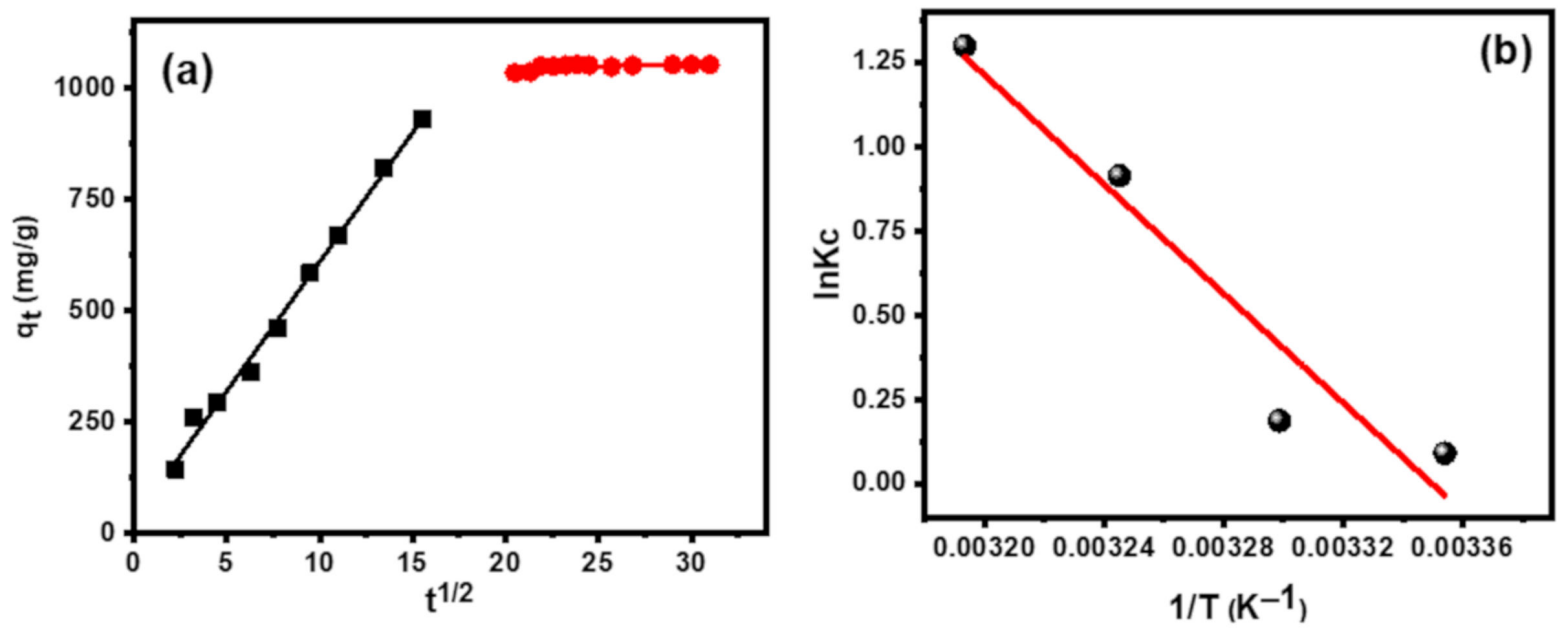
(**a**) Intraparticle diffusion model, and (**b**) thermodynamic analysis.

The obtained results indicated that MB dye adsorption on CT-*g*-PAA/O-ECNFs takes place through the interaction between the active functional groups of CT-*g*-PAA/O-ECNFs and MB molecules. Because the hydrogel network is often porous in nature, it is expected that the pore filling process will contribute to determining the time required for adsorbing the MB dye and reaching the equilibrium state. Therefore, an intraparticle diffusion model was used to verify this effect through the plot between *t*^0.5^ versus adsorption capacity (*q_t_*) as shown in (Figure 11a). The obtained results indicated that the adsorption process was achieved through two steps. The first step was fast and is ascribed to the film formation on the exterior surface of hydrogel network, while the second step was slow and can be ascribed to the intraparticle diffusion process. Although the second step was slow, it was not the monitoring step of the adsorption process, since the straight line does not pass through the origin point.

#### 3.3.5. Thermodynamic Study

Thermodynamic studies provided valuable information about the adsorption process and whether the process was exothermic or endothermic, spontaneous or non-spontaneous. Therefore, the adsorption process was planned at diverse temperatures and of thermodynamic parameters as standard enthalpy change (Δ*H°*), standard free energy change (Δ*G°*), and the standard entropy (Δ*S°*) were determined. The following equations were applied to find the thermodynamic parameters and equilibrium constant (*K_c_*):

(5) ∆G°=−RTlnKc

(6) $$lnK_{c}=\frac{∆S{}^{\circ}}{R}-\frac{∆H{}^{\circ}}{RT}$$

(7) $$K_{c}=\frac{C_{ad}}{C_{rd}}$$

where *R* is the universal gas constant (8.314 J/mol K), *T* is the temperature of adsorption process (K), *K_c_* is the adsorption equilibrium constant, and *C_ad_* and *C_rd_* are the amount of adsorbed dye (mg/L) and amount of residual dye in solution, respectively.

Figure 11b shows a linear plot of 1*/T* versus *lnK_c_* for the adsorption of MB onto CT-*g*-PAA/O-ECNFs at 298, 308, 318, and 328 K, and Table 3 displays the values of *lnK_c_*, Δ*G°*, Δ*H°*, and Δ*S°*.

The negative values of Δ*G°* at different temperature confirmed that the adsorption of MB dye onto CT-*g*-PAA/O-ECNFs was spontaneous and the adsorptive forces for overcoming on potential barrier was sufficient. The positive value of Δ*H°* and Δ*S°* showed that the adsorption of MB dye onto CT-*g*-PAA/O-ECNFs was endothermic and the affinity between MB dye and CT-*g*-PAA/O-ECNFs was good.

#### 3.3.6. Reusability

**Figure 12:**
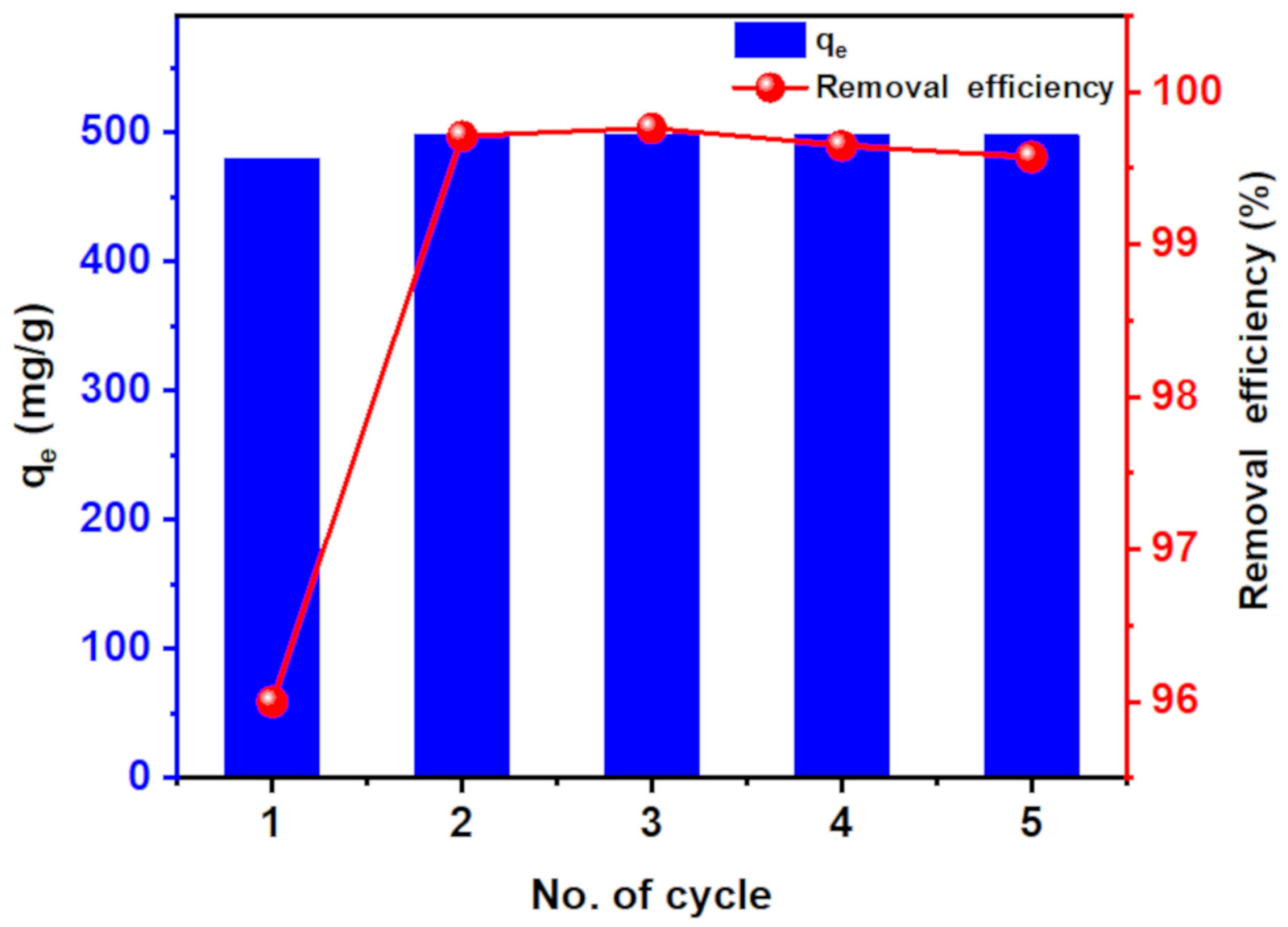
Five reuses of CT-*g*-PAA-O-ECNFs hydrogel nanocomposite.

Reusability of the adsorbent plays a pivotal role in determining its cost. Therefore, the reuse of hydrogel nanocomposite was examined five times for MB dye removal (500 mg/L). The adsorption capability and the removal efficiency for MB dye removal of CT-*g*-PAA/O-ECNFs hydrogel were enhanced after the first cycle and then retained within subsequent four times of reuse (498 mg/g and 99.5%) (Figure 12). The enhancement of its adsorption capacity and removal efficiency after the first reuse cycle can be ascribed to the increase in the size of pores as a result of the swelling process. It is worth mentioning that the hydrogel nanocomposite was easily collected after all cycles of the adsorption process without losing its initial weight. This result clearly indicates the possibility of using the prepared hydrogel nanocomposite as a promising, low cost, and highly effective reusable adsorbent for practical applications in removing pollutants from wastewater.

The comparisons of the maximum adsorption capacity (*q_max_*) and the removal efficiency of the recycled porous hydrogel nanocomposite with reported studies are listed in Table 4 [45,46,47,48,49,50,51,52]. It was obvious that the *q_max_* and the removal efficiency after using of CT-*g*-PAA/O-ECNFs-0.5 for five times at 298 K was much higher than that of other hydrogel composite reported previously, such as poly(vinyl alcohol)/carboxymethyl cellulose/graphene oxide/bentonite (PVA/PCMC/GO/bentonite) [45], xanthan gum-poly(acrylic acid)/reduced graphene oxide (XG-PAA/rGO) hydrogel [49], Magnetic Starch/PVA [50], three-dimensional graphene oxide/β-cyclodextrin/chitosan (3D-GO/CS/β-CD) [51], and xanthan gum-cal-poly(acrylic acid)/oxidized-multi-walled carbon nanotubes (XG-cl-PAA/o-MWCNTs) [52]. The exceptionally high adsorption capacity and reusability of the CT-g-PAA/O-ECNFs hydrogel nanocomposite emerges from the oxygenated functional groups of hydrogels and O-ECNFs, good dispersion of O-ECNFs in hydrogel matrix, and porous structure of the hydrogel nanocomposite, which facilitate the adsorption of MB dye. According to the obtained results, it may be concluded that the CT-*g*-PAA/O-ECNFs hydrogel nanocomposite can be applied as an effective and promising adsorbent for the removal of cationic dyes from polluted water.

## 4. Conclusions

In this study, CT-*g*-PAA/O-ECNFs porous hydrogel nanocomposite was prepared through in situ polymerization process and was utilized as adsorbent for MB dye removal from polluted water. The incorporation of O-ECNFs into CT-*g*-PAA network plays a vital role in monitoring its swelling property. The swelling capacity of CT-*g*-PAA and CT-*g*-PAA/O-ECNFs hydrogel nanocomposites with different ratios of O-ECNFs (0.5–2%) was 249, 40, 22, and 16 g/g, respectively. The adsorption study confirmed that the incorporation of O-ECNFs with 0.5% into hydrogel led to enhancement in its adsorption capacity towards MB dye removal (1095 mg/g). The adsorption capability reached the maximum at pH 7, which enriched the interaction between the adsorbent and the MB molecules. The results of the isotherm study and the adsorption kinetic indicated that the suitable models for describing the process of MB dye adsorption were the Freundlich and PSO models, respectively. Additionally, the calculated thermodynamic parameters displayed that the adsorption process was spontaneous and endothermic in nature, and the affinity between MB molecules and active functional groups of CT-*g*-PAA/O-ECNFs was good. Notably, CT-*g*-PAA/O-ECNFs can be easily collected after adsorption process and regenerated for five cycles with no significant loss in the adsorption capability. Based on the high adsorption capability and the possibility of recycling, the prepared hydrogel nanocomposites are a promising, effective, and highly reusable adsorbent for practical applications in removing pollutants from wastewater with low cost.

## Figures and Tables

**Table 1:** Equations and parameters of non-linear isotherm models of adsorption MB dye onto CT-*g*-PAA/O-ECNFs hydrogel nanocomposite.

Model	Equation	Parameters			*R* ^2^	*χ* ^2^
**Langmuir**	$q_{e}=\frac{Q_{o}K_{L}C_{e}}{1+K_{L}C_{e}}$	*Q_o_*	*K_L_*		0.99349	5.87
		1095.11 ± 28.86	0.0482 ± 0.0116			
**Freundlich**	$q_{e}=K_{f}\;C_{e}^{1/n}$	*K_F_*	*1/n*		0.99358	5.69
		540.93 ± 67.10	0.1108 ± 0.0227			
**Sips**	$q_{e}=\frac{q_{s}\;K_{s}C_{e}{}^{ns}}{1+K_{s}C_{e}{}^{ns}}$	*q_s_*	*K_s_*	*n_s_*	0.9942	5.16
		1231.38 ± 43.07	0.2388 ± 0.0467	0.5292 ± 0.0709		
**D-R**	$q_{e}=q_{o}\;e^{-K_{D-R}\;\varepsilon^{2}}$ $\varepsilon =RTln(1+\frac{1}{C_{e}})$ $E=\frac{1}{\sqrt{2K_{D-R}}}$	*q_o_*	*K_D-R_*	*E*	0.9888	9.80
		1030.39 ± 22.49	159.24 ± 47.57	0.05603 ± 0.0006		

**Table 2:** Equations and parameters of non-linear Kinetic models of MB dye adsorption onto CT-*g*-PAA/O-ECNFs hydrogel nanocomposite. PFO, pseudo-first-order; PSO, pseudo-second-order.

Model	Equation	Parameters				*R* ^2^	*χ* ^2^
**PFO**	$q_{t}=q_{e}\left(1-e^{-K_{1}t}\right)$	*q_t.exp_*		*q_t.cal_*	*K*_1_	0.9790	539.80
		1052.37		1049.33 ± 2.68	0.0096 ± 2.59 × 10^−4^		
**PSO**	$q_{t}=\frac{q_{2}^{2}k_{2}t}{1+q_{e}K_{2}\;t}$			*q_t.cal_*	*K_2_*	0.9987	4.45
				1155.26 ± 15.83	1.38 × 10^−5^ ± 1.82 × 10^−6^		
**Elovich**	$\;q_{t}=\frac{1}{\beta }\ln\left(1+\alpha \beta t\right)$	*α*		*β*		0.9956	17.72
		181.87 ± 131.25		0.00628 ± 8.29 × 10^−4^			
**Intraparticle diffusion**	$q_{t}=K_{id\;}t^{0.5}+C$	*K_id_*_1_	*K_id_*_2_	*C*_1_	*C*_2_	0.9923	--
		58 ± 1.92	1.2 ± 0.426	30.1 ± 17.78	1018.6 ± 10.77		

**Table 3:** Thermodynamic parameters for MB dye adsorption by CT-*g*-PAA/O-ECNFs hydrogel.

*T* (K)	Van’t Hoff Equation	*K_c_*	Δ*G°* (kJ/mol)	Δ*H°* (kJ/mol)	Δ*S°* (kJ/mol)
**298**	y = −8204x + 27.47 *R*^2^ = 0.9348	1.095	−0.2257	68.21 ± 12.35	0.228 ± 0.0408
**308**		1.20	−0.4716		
**318**		2.49	−2.3387		
**328**		3.66	−3.3809		

**Table 4:** Comparison of adsorption capacity and reusability of CT-*g*-PAA-O-ECNFs for MB dye removal with other hydrogel composites.

Adsorbent	*Q_e_* (mg/g)	Regeneration Number/Removal Efficiency (%)	Ref
PVA/PCMC/GO/bentonite	136.56	4/93.4 *	[45]
polyacrylamide/cellulose	326	-	[46]
SA-g-PAA/TiO_2_	2257.36	5/91.4	[47]
Chitosan/montmorillonite	387	-	[48]
XG-cl-PAA/rGO hydrogel	793.6	4/96	[49]
Magnetic Starch/PVA	23.53	8/85	[50]
3D-GO/CS/β-CD	1134	5/75	[51]
XG-cl-PAA/o-MWCNTs	521	4/74.8	[52]
CT-*g*-PAA/O-ECNFs	1095	5/99	This study

* Recalculated removal efficiency.
